# On-farm diversity, use pattern, and conservation of enset (*Ensete ventricosum*) genetic resources in southern Ethiopia

**DOI:** 10.1186/s13002-022-00569-x

**Published:** 2023-01-05

**Authors:** Tesfaye Dilebo, Tileye Feyissa, Zemede Asfaw, Ashagire Zewdu

**Affiliations:** 1 Department of Microbial, Cellular and Molecular Biology, Addis Ababa University, Addis Ababa, Ethiopia; 2 Institute of Biotechnology, Addis Ababa University, Addis Ababa, Ethiopia; 3 Department of Plant Biology and Biodiversity Management, Addis Ababa University, Addis Ababa, Ethiopia; 4 Centre for Food Science and Nutrition, Addis Ababa University, Addis Ababa, Ethiopia

**Keywords:** Abundance, Farmer-named landraces, Interspecific diversity, Landrace richness, On-farm management

## Abstract

**Background:**

Enset is an important source of food and is consumed by about 25 million people as a staple or co-staple food crop mainly in southern parts of Ethiopia. Large numbers of enset landraces exist in different administrative zones of Ethiopia with a wide range of altitudes and agroclimatic zones. However, limited information is available on the diversity, distribution, and utilization pattern corresponding to the diverse ethnolinguistic as well as sociocultural communities of the country. Hence, this study was devised to explore and document the richness of farmers’ tradition and practice on the diversity and distribution of enset landraces on the farm level and selection pattern for different purposes regarding the production, utilization, and conservation of enset genetic resources.

**Methods:**

The study was conducted in four major enset-growing administrative zones of Ethiopia, namely Hadiya, Kembata-Tembaro, Gurage, and Silte. A total of 240 farm households were surveyed using individual interviews, 18 key informant interviews, 36 focus group discussions with 5 participants, and direct on-farm field observations for data collection. Considering that enset has a rich cultural background and indigenous knowledge, ethnobotanical research approach was applied to data collection and analysis. The Shannon–Weaver, Simpson, Pielou, and Jaccard’s similarity indices were used to evaluate the diversity and similarity of the landraces as well as using descriptive statistics in SPSS Ver. 24. Preference in direct matrix ranking was also used to compute and rank the enset landraces most preferred by the people in the context of specific use value in the study area.

**Results:**

A total of 282 farmer-named enset landraces have been identified, with a range from 2 to 32 on individual homegardens. The largest number of landraces was found in the Hadiya Zone (86), while the lowest was scored in the Silte Zone (57). The Shannon diversity index (H') ranged from 3.73 (Silte) to 3.96 (Hadiya). Similarly, landraces revealed a very narrow range of variances in Simpson’s 1-D diversity index, and it ranged from 0.963 (Silte) to 0.978 (Hadiya). Likewise, the similarity index ranged from 0.24 to 0.73 sharing 16–47 landraces in common. Of the 282 landraces, 210 (74.5%) were recorded in more than one zones, whereas 72 (25.5%) had narrow distribution being restricted to a single zone.

**Conclusions:**

Farmers have established long-term practices and experiences in cultivation, utilization, and conservation of a diverse group of enset landraces to fill their domestic and market purposes in each zone. The variation is likely to be related to agroclimatic differences, ethnicity factors, food cultures, and historical backgrounds. Therefore, to facilitate on-farm conservation as well as sustainable utilization of the enset genetic resources, farmers need to be supported by different stakeholders for all their worth and also in crop improvement programs.

## Introduction

Enset [*Ensete ventricosum* (Welw.) Cheesman] is a large perennial monocarpic herbaceous plant, similar to the banana in form, in the family *Musaceae* within the monocot order of *Zingiberales* [1]. *E. ventricosum* is domesticated, and the corm (short underground stem) and pseudostem (thick and soft midrib) are processed and consumed as a staple and co-staple food in the south and southwestern parts of Ethiopia [2]. Enset is distributed at altitudes between 1500 and 3100 masl and it is chiefly propagated vegetatively [3]. It is noted for its tolerance to environmental fluctuations, storability, and for its multiple uses that play a pivotal role in preventing famine [4, 5]. Moreover, enset in Ethiopia is arguably a very important crop contributing to food security and rural livelihoods for about 25% of the Country's population [2, 6, 7] with diverse ethnic and cultural backgrounds. Ethiopia is both the center of origin and center of diversity for enset and many other crops [8]. This diversity is maintained on-farm by farmers who also continue to diversify it through exchanging, sharing, and purchasing seedlings for cultivation. Genetic diversity for farmers means varietal diversity, which they can differentiate on the basis of agromorphological traits, phenological attributes, product quality, post-harvest characteristics, and differential adaptive performance under abiotic and biotic stresses [9–11].

Farmers have managed the diversity of enset landraces for centuries with limited or no research influences from outside [12, 13] being managed almost purely by indigenous knowledge and skills. Numerous landraces are grown for different uses and for the cultural requirements of the people at different sites of cultivation [14, 15]. Some prior studies indicate that numerous enset cultivars were identified in the south and southwest parts of Ethiopia and the observed genetic diversity in cultivated enset in a particular area appears to be related to the agroclimatic variation, the extent of enset cultivation, and the culture and distribution pattern of the different ethnic groups including the Gurage, Hadiya, Kembata, Silte, Wolaita, Dawuro, Ari, Kefa, Sheko, and many others [13, 16–19]. Farmers select enset landraces based on the quality and quantity of food products (the fermented scrapings known as *qocho*, the juice from the scrapings known as *bulla,* and the boiled corm known as *amicho*), rate of maturation, disease and drought tolerance, forage quality, medicinal value, ease of scraping, quality of corm, and productivity [17, 20, 21].

Understanding the diversity and distribution of enset is crucial for sustainably managing genetic resources and crop improvement efforts. Yemataw and co-workers [13] showed that the abundance and distribution of enset landraces in their study area exhibited substantial variances based on their use value and local naming and classification system. Some landraces, especially those with attributes of better quantity and quality of products, have a wider distribution both within and between zones.

Shigeta [22] described that different enset landraces are recognized in different growing areas of Ethiopia, the only country where it is grown as a food crop, and are being grown in mixtures. Each enset landrace as identified by farmers has its name that is commonly used across the areas inhabited by people that speak the same language (with possible dialects/cognate names within some languages) but is sometimes shared by adjacent ethnic groups [16, 17, 23]. Farmers differentiate one landrace from the other phenotypically by looking at the color of the petiole, midrib, leaf sheath, angle of leaf orientation, size, and color of leaves, and circumference and length of pseudostem [16, 17, 24, 25]. Hence, vernacular names are often descriptive and reflect variations of landraces in places of origin, morphology, as well as agronomic and cooking characteristics [12, 26]. However, in some cases there are similar landraces known by different vernaculars and there are also different landraces known by similar vernaculars and with similar phenotypic appearance [23, 27].

The high genetic diversity of enset warrants conservation, as it provides resilience to the enset farming system and thus food security for farming communities [13, 16, 18]. Enset plays a crucial economic role, providing higher production under low input conditions compared to other crops in Ethiopia [28–30]. It is a multipurpose crop and nearly every part of the plant has some sort of use as food and non-food [2, 31]. Farmers often say that enset is their food, their cloth, their house, their bed, their cattle feed, and their plate [2]. The major food types obtained from enset are *qocho*, *bulla*, and *amicho*. Furthermore, some enset varieties are used traditionally to cure bone fractures, birth problems, and diarrhea in humans [16, 25, 32].

Enset landraces are grown in homegardens with different local names and often with wide distribution and varietal diversity with implications to genetic diversity. For the sustainable utilization and on-farm conservation of its genetic resources as well as future improvement of the crop, understanding the sociocultural, ethnobotanical knowledge, farmers’ selection criteria, and retention practices of enset landrace diversity in different ethnolinguistic communities is vital. However, limited documentation (e.g., [16, 17], and [29]) is available concerning the on-farm varietal diversity, its distribution, and the pattern of uses in different zones or ethnic groups. Due to this, the present study helps to fill the knowledge gap concerning farmers’ traditional practice on enset cultivation and utilization in Hadiya, Kembata-Tembaro, Gurage, and Silte zones, the major enset production areas in southern Ethiopia. Therefore, this study aimed at documenting the richness of farmers’ ecological knowledge, tradition, and practices regarding the diversity and distribution of enset landraces on the farm level and the naming and selection criteria for different purposes concerning the production, utilization, and conservation of genetic resources.

## Materials and methods

### The study area and site selection

**Fig. 1:**
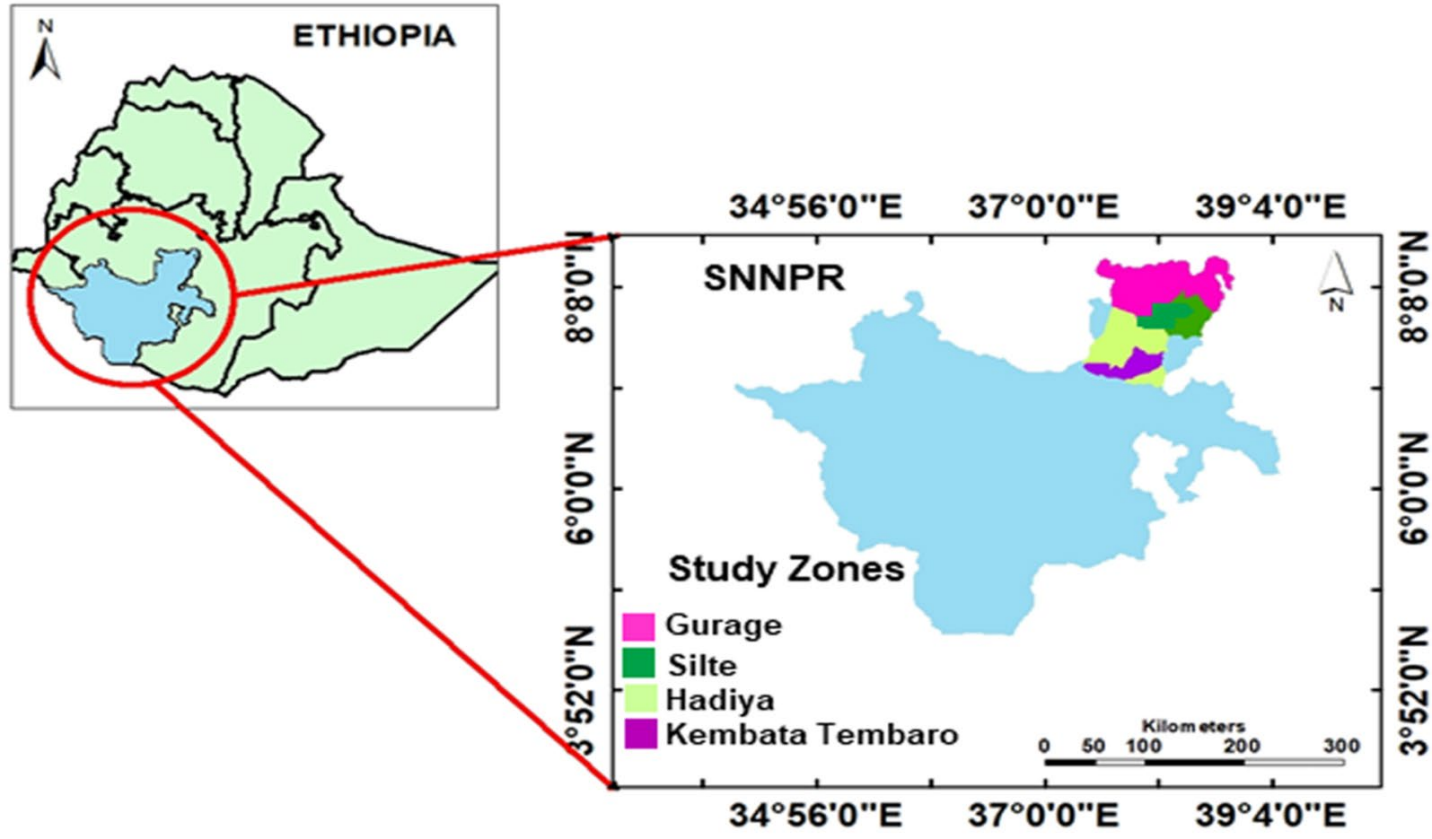
Location of the Southern Nations Nationalities and Peoples' Region (SNNPR) in the map of Ethiopia (left) and the four study zones in the SNNPR (right)

The study was conducted in four enset-growing administrative zones, namely Hadiya, Kembata-Tembaro, Gurage, and Silte of southern Ethiopia (Fig. 1). The zones are basically distinguished by distinct languages, cultural background, and farming systems and also named based on the name of the predominant ethnic group for that administrative location. The Hadiya and Kembata-Tembaro peoples speak a Cushitic language family, while the Gurage and Silte peoples belong to groups speaking the Semitic language family. Generally, the study zones are located between the great Ethiopian Rift Valley and Gibe-Omo River system and are bordered by the Oromia region to the north and east, and with Wolaita zone in the south. The zones are structured into different *woredas*, which are further organized into *kebeles* (the lowest administrative units in Ethiopia). The study *woredas* and *kebeles* were selected from each administrative zone based on enset diversity where prior information was obtained from the departments of agriculture of the respective zones and *woredas* (Table 1).

**Table 1:** Description of the studied areas, number of respondents, and altitude ranges

Administrative zone (major language family spoken)	Study woredas	Sampled kebeles	Number of respondents per kebeles	Altitude ranges (m)
Kembata-T* (Cushitic)	Doyogena	Murasa	7	2145–2255
		Hawora-Arara	6	2335–2565
		Serera	7	2650–2822
	Angacha	Wasera	7	2550–2675
		Qerekecho	7	2225–2360
		Funamura	6	2150–2220
	Damboya	Dato	6	2680–2760
		Kazala	7	2250–2470
		Bonga	7	2300–2435
Hadiya (Cushitic)	Misha	Tulla	7	2675 – 2915
		Dengawora-S*	7	2567–2784
		Semenwasgebeta	6	2250–2465
	Lemmo	Shurmo-Dacho	7	2200–2350
		Dijo-Demala	7	2201–2418
		Lisana	6	2145–2250
	Dunna	Somicho	7	2475–2676
		Woramera	6	2300–2565
		Qenqicho	7	2435–2550
Gurage (Semitic)	Endegegn	Shewora	7	2210–2345
		Wolecho	7	2165–2455
		Zigez	6	2275–2335
	Gumer	Esen -Adengez	6	2635–2785
		Gura-Fezer	7	2695–2750
		Qebul	7	2570–2790
	Enamor-Ener	Agata	7	2235–2560
		Kochira	7	2195–2275
		Jatu	6	1850–2230
Silte )(Semitic)	Mirab A*	Willo	6	2485–2560
		Woger-gunjubul	7	2445–2585
		Mugo	7	2850–3195
	Misraq A*	Awerad	7	2265–2310
		Semerdin-D*	6	2220–2385
		Gomoro—Bucha	7	2315–2425
	Alicho W*	Abzena hulat	6	2775–3170
		Kutere	7	2450–2575
		Bune saqemo	7	2550–2680

A* = Azernet, D* = Derawote, S* = Segamo, T* = Tembaro, W* = Weriro

### Sampling technique and sample size

For this study, a multistage sampling method was performed for the selection of individual enset-growing farmers in each zone. Of the four administrative zones, 12 *woredas* (three from each zone) were selected purposefully based on enset frequency of occurrence and production level. From each *woreda*, three *kebeles* were also chosen purposefully according to the information obtained from the agricultural office of each *woreda*. Therefore, a total of 36 *kebeles* were selected for data collection. From each *kebele*, six to seven individual farmers were selected randomly that make a total of 240 households (60 household heads from each zone) in the whole study sites.

### Ethical consideration

The Microbial, Cellular, and Molecular Biology Department (Addis Ababa University) initially reviewed the study proposal. Following the approval, a supporting letter was written to the zonal administrative offices of the study area adhering to the existing national guidelines. As a result, each district/*woreda* official was informed of the study's objectives and wrote supporting letters to notify their respective kebele administrative offices. After obtaining the *kebele* leaders' permission, the investigator, local elders, and the agricultural extension workers of each *kebele* had a comprehensive discussion about the study's objectives and a schedule for the fieldwork and interview sessions. Following verbal informed consent of each informant, interviews and discussions were conducted to gather indigenous knowledge (non-clinical sample study) held by knowledgeable informed volunteers and participants about the on-farm diversity, use patterns, and traditional management practice of the enset crop.

### Data collection

Both primary and secondary data collection methods were conducted to assess and document farmers’ local knowledge regarding on-farm diversity, distribution, and utilization of enset crops in the study area. Two rounds of data collection and field observation were conducted (the first round from June to September 2019 for preliminary observation and conducting the majority of interviews, and the second round from January to March 2020 for direct observation of field activities like planting and transplanting).

To develop semi-structured interviews, different kinds of discussion were conducted initially with three to four elder enset farmers in each zone to generate needed information to be collected in the study area. In-depth individual interviews were conducted together with trained agricultural extension workers, who are working closely with the communities in the respective selected *kebeles* in local languages (Hadiya, Kembata, Gurage, and Silte languages using a translator) and in the Amharic official language. The principal investigator can also communicate and understand three of the above-listed local languages; hence this made our work easier and the communication very smooth.

The farmers were also asked about their perception of names and naming systems. To obtain the detailed local knowledge of farmers in each zone, 4–5 key informants were also selected based on prior information obtained from *woreda* agricultural experts, agricultural extension workers of *kebeles*, elderly farmers, and local leaders. For the focus group discussions, from each of the selected *woredas,* about five participants were involved together with the members of the local administration, community elders, agricultural extension workers, and other members of participating communities. Additional data were also collected through, preference in direct matrix ranking by involving 12 key informants (three from each zone). Secondary data were also reviewed from the reports of the agricultural office of each zone, different books, research articles, and journals.

### Data analysis

All listed landraces throughout the collection sites were checked for known synonyms or local names that refer to the same or different landraces in each study zone and *woreda* (district) with the help of knowledgeable senior farmers. Moreover, some minor dialect variations in naming landraces within the same ethnic group were not considered different and were disregarded in landrace authentication. However, landraces having the same names, but originating from different ethnic groups or zones, were documented as its. The collected survey data were analyzed using descriptive statistics (frequency, percentages, and average) in SPSS Ver. 24. The landrace richness, distribution, and abundance per homegarden were also calculated using Microsoft Excel 2010. Richness was computed to show the total number of landraces per homegarden based on data recorded in each administrative zone as this is a simple applicable biodiversity index to use and compare diversity in enset landraces. Abundance was determined as the total number of individual enset plants of each landrace per homegarden. Preference in direct matrix ranking was conducted to analyze the most preferred enset landraces, in the context of the four specific use values for the seven enset landraces. Twelve key informants participated in the arrangement of the values by giving the most favored enset landraces a score of 10, the least preferred enset a score of 1, 0 for the uses not known, and the others a score that fell somewhere in between. Based on the total scores obtained for each landrace, these values were then summed for all respondents and ranked.

Diversity and similarity indices of species can be quantified in different ways. In this study, the diversity indices were calculated from the number of landraces existing in 60 farmers’ homegardens within each zone. The Shannon and Weaver [33] and Simpson Index [34] was used to evaluate the landraces diversity. Both of them are widely used tools as a measure of heterogeneity [35], and these were calculated for all sample zones to explore enset diversity. Shannon–Weaver diversity index is the most popular measure of species diversity because it accounts both for species richness (numbers) and evenness, and it is not affected by sample size [36]. The resulting index is high when the relative abundance of the different species or landraces in the sample is even and is low when a few species or landraces are more abundant than the others. It was calculated using the formula: H′ =  − Σ pi ln pi[35], where pi is the proportional abundance of the *i*th landrace.

Even though Shannon’s index takes into account the evenness of the abundance of landraces, evenness (equitability) can also be computed separately. It is a measure of the proportion of the observed diversity for the maximum diversity expected and was calculated through the Pielou index [37] as the ratio, E = H′/H′max = H′/lnS, where: E is the evenness (equity) index; H′ = diversity; H’max is a maximum diversity; lnS, in which S refers to the number of landraces in each zone. The higher the value of E, the more even the species is in their distribution within the community or the plots. Similarly, the higher the value of H’, the more diverse the community or the plot is. A high evenness, resulting from all cultivars (landraces) having an equal abundance, is normally equivalent to high diversity [35].

Simpson’s diversity index (**D**) is a measure of diversity. It measures the probability that two individuals randomly selected from an area will belong to the same species [34] and hence, as D increases, diversity decreases. The index was, therefore, transformed as 1-D so that greater diversity corresponds to higher values: The formula for calculating D is presented as:

$D = \frac{{\sum {ni\left( {ni - 1} \right)} }}{{N\left( {N - 1} \right)}}$, or (1-*D*) = 1 − {Σ*n* (*n* − 1)/*N* (*N* − 1)} The value of this index ranges between 0 and 1; the greater the value, the greater the diversity, 1 represents infinite diversity and 0, no diversity. The index was computed for all study zones.

Sorenson similarity index was employed to assess differentiation or beta (*b*) diversity [35], and it compares the similarity of species (landrace) diversity among the study zones. The expected variation in landrace composition that exists between the study zones was computed using Sorenson’s similarity coefficient (Cs) [38].

$$Cs = 2J/a + b$$

where *a* is the number of landraces at zone A, *b* is the number of landraces at zone B, and *J* is the number of landraces common to both locations. Sorenson's similarity coefficient ranges in value from 0 (no similarity) to 1 (complete similarity).

## Results and discussion

### Socioeconomic characteristics of respondent households

A sample of respondents on socioeconomic characteristics is described in Table 2. Among the respondents, 82.1% of families were male-headed households, while only 17.9% were female-headed households. About 50.4% of the heads of households were between the ages of 45 and 65, while 25.4% of the respondents were over 65. Approximately 41% of respondents were illiterate, whereas 28.8% had informal education and could read and write. However, 51% of the respondents overall in the studied administrative zones who participated in the interview were female. They are knowledgeable enset cultivators who have a great deal of knowledge about planting, managing in the field, harvesting, and using enset products. They rely on enset products for most of their food needs, medical requirements, needs for fodder, and environments. They also gain benefits from the rich ecosystem of goods and services created by the enset agrosystem.

**Table 2:** Socioeconomic characteristics of respondent households

Variable	Category				Zone					
		K-T		Had		Gur		Sil		
		*N*	%	*N*	%	*N*	%	*N*	%	Mean%
Sex of HH	Male	49	81.67	51	85	47	78.33	50	83.33	82.08
	Female	11	18.33	9	15	13	21.67	10	16.67	17.92
Age of HH	< 45	16	26.67	16	26.67	14	23.33	12	20	24.17
	45–65	30	50	28	46.67	33	55	30	50	50.42
	> 65	14	23.33	16	26.67	13	21.67	18	30	25.42
Education level	Illiterate	23	38.33	24	40	25	41.67	26	43.33	40.83
	Read and write	16	26.67	15	25	19	31.67	19	31.67	28.75
	Primary	13	21.67	13	21.67	12	20	11	18.33	20.42
	Secondary	8	13.33	9	15	4	6.67	4	6.67	10.42

HH = household, K-T = Kembata-Tembaro, Had = Hadiya, Gur = Gurage, Sil = Silte, *N* = number of respondents

### Extent of richness and diversity of enset landraces

In this study, we identified and recorded 282 locally named enset landraces in the Hadiya, Kembata-Tembaro, Gurage, and Silte zones of southern Ethiopia. Enset growers can easily distinguish one enset landrace from the other by observing the external (leaf structure, size, orientation, midrib color, and other) and internal features (leaf and midrib anatomy and fiber structure) of the enset plants, and they give distinct vernacular names for each landrace. Each local farmer in the studied area was observed cultivating a diverse of enset landraces in his or her homegarden, which shows a considerable variation in the number of enset landraces on individual homegardens. It ranges from two to thirty-two in this study (Table 2). According to farmers’ knowledge of local names: 86 enset landraces from Hadiya, 73 from Kembata-Tembaro, 66 from Gurage, and 57 from Silte were recorded. The highest and lowest number of landraces per homegarden was documented in Hadiya and Silte zones, respectively (Table 3). In comparison with earlier reports, a relatively larger number of landraces have been identified in this study. The literature shows that Tsegaye [17] recorded 146 different enset landraces including 59 from Hadiya, 55 from Wolaita, and 52 from Sidama while Negash [16] reported the same total number including 65 from Kefa-Sheka, 30 from Sidama, 45 from Hadiya and 6 from Wolaita. Likewise, Birmeta [18] described 111 enset landraces from nine enset-growing localities of Ethiopia that contrasted with the findings of the present study as in some other previous studies. For instance, Yemataw et al. [13] and Zeberga et al*.* [19] described the same numbers of (312) different enset landraces from eight ethnic groups, out of these 69 from Silte, 66 from Kembata-Tembaro, 63 from Gurage, and 51 from Hadiya. Furthermore, Yemataw et al*.* [24], who described 218 different enset landraces from seven zones, came up with 59 landraces from Hadiya, 43 from Kembata, 41 from Dawuro, 39 from Wolaita, 34 from Gamo Goffa, 31 from Gurage, and 30 from Sidama. Some of these values are slightly comparable to the findings of the present study but such records are impossible to make a direct comparison of the number of enset landrace diversity with the results of the current study due to variations in the method and size of the sampling area. However, in most cases, the richness of enset landraces recorded in the current study is far higher than the reports of the previous studies which is likely to be related to the rigor and intensity (including the sampling frame) as well as the knowledge of the men and women informants that participated in the present study. The number of enset landraces in the present study could be attributed to the technique of sampling, the area the study covered, and the nature of the agroecological condition of the study area, which embraces midland and highland that is suitable for enset cultivation. Moreover, the study zones like Hadiya are bordered by all the other study zones, so the exchange and earning of suckers are common traditions among farmers. In the same manner, Tsegaye [17] and Yemataw et al. [24] stated that the exchange of enset landraces from the neighboring ethnic groups perhaps contributed to the richness of enset landrace diversity in Ethiopia.

**Table 3:** Enset landrace diversity in the four administrative zones, richness, Simpson (1-D), Shannon (H') diversity indices, and evenness (E)

Zone	Richness (%)	Min^a^	Max^b^	Mean^c^	Unique^d^	1- D	H'	E
Hadiya	86 (30.5)	3	32	10.23	22	0.978	3.96	0.89
K-T*	73 (25.9)	3	19	8.71	26	0.976	3.88	0.90
Gurage	66 (23.4)	4	24	9.52	14	0.975	3.83	0.91
Silte	57 (20.2)	2	22	8.24	10	0.963	3.73	0.92

* = Kembata-Tembaro, a = Minimum richness, b = Maximum richness, c = Mean richness/homegarden, d = Number of unique landraces

The Shannon diversity index (H′) ranged from 3.73 (Silte) to 3.96 (Hadiya), this signifies the existence of a high richness of enset landraces in the study zones. Even though zones varied in richness, they revealed a very narrow range of variances in Simpson’s 1-D and evenness indices. The Simpson’s 1-D ranged from 0.963 (Silte) to 0.978 (Hadiya) and evenness indices ranged from 0.89 to 0.92. All these results specify the presence of high enset landraces diversity in these four zones (Table 3). This finding is in line with earlier reports [13, 19]. According to [39], the value of a diversity index increases when both richness and evenness increase and is maximized when all species are nearly equally abundant. In biodiversity studies, Shannon diversity indices (H′) typical values range between 1.5 and 3.5 and the index is rarely greater than 4 [40]. The higher the value of H′, the more diverse the communities, and the Shannon index increases as both the richness and evenness of the communities increase.

### Similarities and differences of enset landraces diversity among zones

The similarity among pairs of zones (taking two zones at a time) concerning farmers-named landraces was evaluated using Sorenson’s similarity index (Table 4). Generally, the similarity index ranged from 0.24 to 0.73, and the number of commonly shared landraces varied from 16 to 47. Hadiya and Kembata-Tembaro were the most similar zones, followed by Gurage and Silte about enset landraces (Table 4). Hadiya also shared 38 and 35 enset landraces with Gurage and Silte zones, respectively. This high sharing of enset landraces among zones may be due to sociocultural and linguistic similarities, and geographical locations. For instance, Hadiya is bordered by all study zones, so the informal exchange of enset suckers from the adjacent zones possibly contributed to the highest similarity of enset landrace diversity among zones in the present study. This agrees with the work of [19] and [24], who reported the existence of a high amount of sharing similar enset landraces among Hadiya and Kembata, Gurage and Silte, and Wolaita and Dawuro zones of Ethiopia. On the other hand, pairs of zones with relatively least similarity were Kembata-Tembaro and Silte, and Gurage and Kembata-Tembaro 0.24 and 0.25 for each pair, respectively. This may be due to the geographical distance between the two zones and also variations in sociocultural factors.

**Table 4:** Enset landraces shared (bold) and Sorensen similarity indices between pairs of zones

Zone	Hadiya	K–T*	Gurage	Silte
Hadiya		**47**	**38**	**35**
K-T*	0.59		**17**	**16**
Gurage	0.50	0.24		**45**
Silte	0.49	0.25	0.73	

* = Kembata-Tembaro

### Distribution and abundance of enset landraces

Distribution of the enset landraces throughout the study sites varied across zones. Out of 282 enset landraces recorded, 15 (5.3%) were widely distributed in all four zones. These were *Agade*, *Astara*, *Bededete*/*Badade*, *Gimbo*/*Gimbuwa*, *Heniwa*/*Hiniba*/*Enba*, *Kasete*, *Manduluqa*/*Mande*, *Mariye*, *Merza*, *Mesmesia*, *Moche*, *Separa*/*Sebera*, *Torora/Xorore*, *Weshemeja* and *Zobira* (Table 4). Similarly, 33 (11.7%) farmers’ named enset landraces were commonly cultivated and found in three (Hadiya, Gurage, and Silte) out of four zones (Table 4). Likewise, 72 (25.5%) of the enset landraces had a narrow distribution and were specific to a single zone (Table 4). But the remaining 210 (74.5%) were recorded in more than one administrative zone. The finding of this study was in line with the previous study of [17, 19], and [24] from the same or different zones in Ethiopia.

The abundance of enset landraces also differed among the study zones in addition to their distribution. Few enset landraces such as *Gimbo*, *Hiniba,* and *Separa* were relatively high in abundance at all four study zones. *Agade*, *Bedededa,* and *Zobira* were also other most frequent enset landraces in three out of the four zones (Table 5). Some landraces were well encountered in two zones but virtually absent from the other study zones. For example, *Sisqella* and *Gishira* were the most abundant landraces of the enset homegardens visited in Hadiya and Kembata-Tembaro zones but were almost absent or rare in other zones. Moreover, some landraces such as *Abatemerza*, *Degomerza*, *Dirbo*, and *Unjame* in Kembata-Tembaro, *Amerate* and *Lemat* in Gurage, *Shewrad* in Silte, *Disho,* and *Bequcho* in Hadiya zones were dominant but outside these zones, they were found with a low abundance. A similar observation was reported by [13] and [19], they indicated that landrace *Agade* in Silte, *Amerate* in Gurage, *Shododenia* in Dawuro, and *Addo* and *Genticha* in Sidama encountered a high local abundance at each studied zones. This may be due to the environmental adaptability of the landraces or/and different attributes of farmers. Negash [16] and Tsegaye [17] also reported that enset landrace diversity and distribution were influenced by factors such as household resources, cultural background, population pressure, and agroecology. Enset landraces, namely *Manduluqa*, *Mariye*, *Mesmesia*, *Moche,* and *Torora*, described in this study were found in a limited number of homegardens but widely spread in each zone. In the same manner, [13] and [19] indicated that household features, the distance between locations, and ethnic preference contribute to the landrace diversity and abundance.

**Table 5:** List of farmers-named landraces and their richness in the four administrative zones

No.	Hadiya	*N*	K-T*	*N*	Gurage	*N*	Silte	*N*
1	*Addo*	2	*Abatmerza*	55	*Agade*	51	*Agade*	59
2	*Agade*	38	*Agade*	6	*Agoregure*	11	*Agermir*	12
3	*Alabite*	3	*Aganche*	8	*Ahiro*	18	*Ahiro*	31
4	*Anchire*	5	*Arke*	4	*Amerate*	49	*Ameret*	6
5	*Arke*	2	*Ashure*	26	*Ankufuye*	28	*Ankufaye*	8
6	*Astara*	21	*Astara*	8	*Ashaqit*	4	*Ashaqit*	6
7	*Awunada*	12	*Ayase*	15	*Astara*	42	*Astara*	28
8	*Banko*	2	*Bededed*	9	*Awunad*	6	*Awunade*	7
9	*Bedededa*	32	*Banko*	12	*Aywogna*	5	*Aywongna*	29
10	*Beneje*	18	*Cherquwa*	11	*Bededet*	37	*Bededet*	36
11	*Bequcho*	6	*Danxia*	7	*Benezhe*	32	*Manduluqe*	3
12	*Beshiqiye*	3	*Degomerza*	39	*Bezeria*	23	*Beneje*	30
13	*Bezeriya*	4	*Dereqeta*	8	*Bitena*	3	*Bezeria*	4
14	*Birwesa*	3	*Derga*	6	*Bossora*	21	*Bossora*	16
15	*Boicho*	12	Dirbo-n^*^	12	*Chehoyet*	8	*Bushawesse*	4
16	*Boshosha*	2	*Dirbo-qey*	38	*Dare*	26	*Dem-worad*	11
17	*Danxia*	6	*Disho*	21	*Demyetertnech*	7	*Deriye*	12
18	*Dego*	31	*Uskuruz*	14	*Demyetertqey*	4	*Ferezeye*	6
19	*Dirbo*	21	*Etene*	29	*Egendye*	26	*Fenqo*	3
20	*Disho*	39	*Fechache*	6	*Enba*	38	*Fugnaqir*	2
21	*Egandiya*	6	*Felegede*	4	*Fenqo*	4	*Garado*	6
22	*Etine*	11	*Fello*	3	*Ferezeya*	17	*Guariye*	31
23	*Fechecha*	4	*Ferchase*	9	*Gazner*	8	*Gefate*	3
24	*Fello*	2	*Gagabo*	6	*Gegered*	11	*Gimbo*	41
25	*Feraziya*	3	*Gimbuwa*	39	*Gimbuwa*	28	*Gudero*	6
26	*Gagabo*	2	*Ginawa*	11	*Ginad*	6	*Hanzana*	5
27	*Gariya*	25	*Ginjona*	13	*Gozoda*	12	*Hiniba*	39
28	*Gimbo*	57	*Gishira*	29	*Guarye*	24	*Kaset*	11
29	*Ginjowona*	2	*Guderete*	3	*Gumbura*	3	*Kembat*	12
30	*Gishira*	38	*Gomorsa*	6	*Hanzana*	12	*Kemele*	2
31	*Gomorsa*	5	*Gunze*	3	*Kanchewa*	8	*Kombotir*	4
32	*Gozoda*	4	*Hargema*	5	*Kaset*	9	*Megribe*	3
33	*Gudere*	8	*Hella*	22	*Kebere*	3	*Mariye*	6
34	*Hanazana*	7	*Heniwa*	29	*Kembat*	11	*Merza*	3
35	*Haqucho*	3	*Keset*	4	*Kemele*	4	*Mesmesia*	2
36	*Hayiwona*	29	*Ketane*	2	*Kemota*	2	*Moche*	8
37	*Hella*	24	*Korbo*	2	*Kona*	5	*Nechewo*	5
38	*Hiniba*	41	*Lenbona*	3	*Lemat*	22	*Orad*	6
39	*Hyro*	8	*Leqeqa*	28	*Manduluqe*	2	*Qeshqeshe*	4
40	*Jegirada*	7	*Lokande*	5	*Mariye*	5	*Qiniware*	26
41	*Kaseta*	12	*Manduluqa*	12	*Merza*	4	*Separa*	38
42	*Kekera*	9	*Mariye*	18	*Mesmesia*	7	*Sherafire*	12
43	*Kerqere*	2	*Mesmesa*	15	*Mishirad*	3	*Shewrad*	15
44	*Korina*	8	*Moche*	9	*Moche*	6	*Shigez*	4
45	*Lechebo*	5	*Morala*	3	*Nechewa*	21	*Shireteye*	31
46	*Lendwese*	3	*Mutite*	3	*Oniya*	8	*Sino*	12
47	*Leqeqa*	13	*Nejawro*	2	*Oret*	24	*Sisqella*	2
48	*Lokanda*	6	*Oniya*	21	*Qeshqeshe*	6	*Tegeded*	6
49	*Manduluqa*	3	*Qeqile-nech*	12	*Qibnare*	39	*Tem-wese*	3
50	*Mariye*	11	*Qeqile-qey*	16	*Separa*	42	*Torora*	5
51	*Meqelwesa*	18	*Qerqere*	5	*Shewatia*	6	*Wonade*	9
52	*Merza*	34	*Qorate*	2	*Shewora*	5	*Woshemaja*	6
53	*Mesmesia*	18	*Quina*	22	*Shireteye*	29	*Yekechere*	2
54	*Moche*	25	*Sebera*	37	*Sisasir*	3	*Yetibare*	2
55	*Mutite*	3	*Shate*	2	*Tegeded*	18	*Zegizik*	2
56	*Nechewo*	7	*Shelleqe*	16	*Tereye*	8	*Zerbededet*	9
57	*Oniya*	22	*Sinera*	4	*Torora*	7	*Zobir*	28
58	*Orada*	11	*Sisqella nech*	44	*Wonadia*	11		
59	*Ossosa*	4	*Sisqella tikur*	12	*Woshemadia*	6		
60	*Qebere*	7	*Sorpie*	8	*Yeqesewa*	18		
61	*Qenchewa*	2	*Unjame*	41	*Yeshirafire*	12		
62	*Qeshqeshe*	6	*W’ea*	12	*Yeshiraqinqe*	15		
63	*Qeteqeta*	2	*Wachiso*	7	*Zegirad*	9		
64	*Qiniwara*	26	*Wellanche*	5	*Zerbededet*	12		
65	*Qombotira*	15	*Weshemeja*	2	*Zobir nech*	3		
66	*Quiena*	9	*Woio woe*	3	*Zobir qey*	27		
67	*Separa*	43	*Wolegella*	8				
68	*Shate*	29	*Wongorate*	3				
69	*Shelleqe*	3	*Xebare*	22				
70	*Shereqa*	2	*Xessa*	29				
71	*Shewora*	7	*Xorore*	27				
72	*Shirafire*	14	*Zinke*	4				
73	*Sinera*	3	*Zobira*	6				
74	*Sisqella*	53						
75	*Soqido*	18						
76	*Suwandiya*	2						
77	*Tegeded*	6						
78	*Unjame*	19						
79	*Uskurusa*	5						
80	*Wea*	3						
81	*Wonade*	6						
82	*Woshamaja*	7						
83	*Xessa*	13						
84	*Xiggo*	9						
85	*Xorore*	27						
86	*Zobira*	39						

*N* = Number of respondents who are growing the above-listed landraces, K-T^*^ = Kembata-Tembaro

### Diverse local names of the enset landraces among zones

**Fig. 2:**
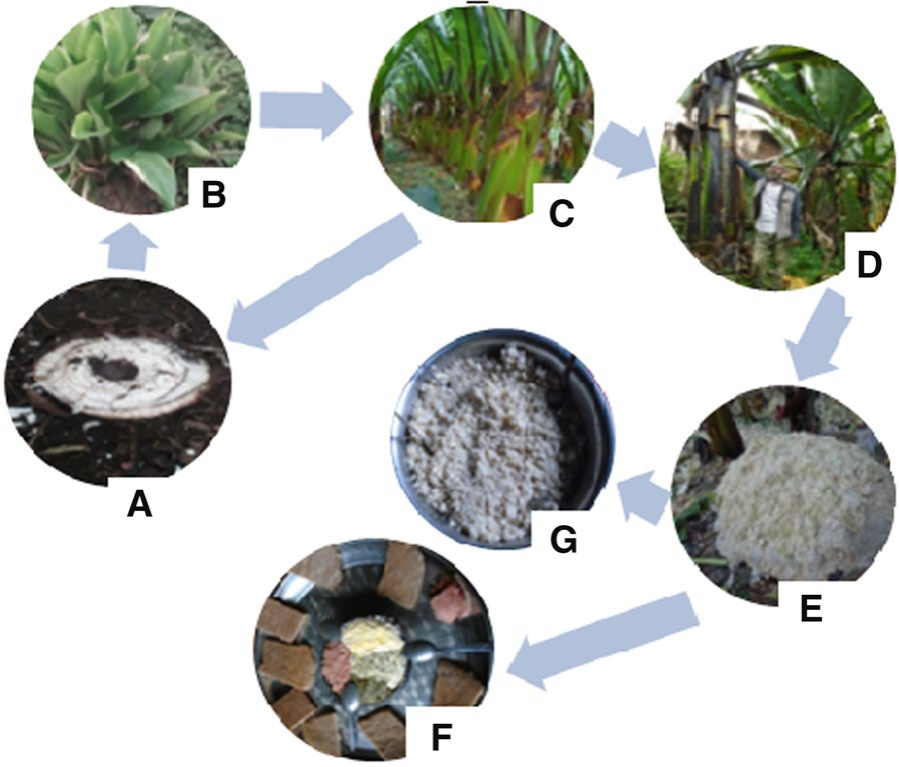
Examples of the main enset propagation stages and its end products: **a** mother corm; **b** multiple suckers from mother corm; **c** young enset ready to give mother corm and transplanted to permanent field; **d** matured enset; **e** mass of processed product; **f** and **g** example of dishes that prepared from the primary products

The local names of enset (*Ensete ventricosum*) and its different growth stages vary from one ethnic group to another. Enset is called *wessa* in Hadiya and Kembata-Tembaro, *wesse* in Silte, and a*set* in Gurage. Moreover, each growth (transplanting) stage has a distinct name by which it is identified. The Hadiya and Kembata-Tembaro farmers share almost the same local names for all sucker stages. These are known as *dubbo*, *simma*, *ero*/*kiniba,* and *balwesa*, but in Silte 1 -and 2-year-old suckers are called *bosho* and *daporo*, respectively, and the other two stages are nearly similar to the Hadiya and Kembata-Tembaro zones (Fig. 2a–d). In Gurage, 1-year-old sucker is *fonfo*, but the second and third stages are named the same as other studied zones.

According to the interviewed farmers, the same enset landraces are sometimes known by different names in different administrative zones (Table 6). In this study, 11 farmer-named landraces identified with the help of knowledgeable farmers in each zone indicated that the same enset landraces were known by different names in the other studied zones (Table 6). The role of knowledgeable men and women enset farmers was so critical in this research since they are experts in the landrace identification and description of ethnobotanical methodology. The landrace names given by enset farmers mostly reveal distinct morphological appearances or other culinary characteristics such as taste or use values (data not shown). Each ethnic group has its series of local names for enset landraces. For example, the landrace *Shate* in Hadiya, and *Shirteye* in silte and Gurage are the same landrace with different local names often representing the bitter-tasting characteristics of all its parts. Enset landrace *Xiggo* in Hadiya, called *Qeqile-nech* in Kembata-Tembaro, is well known to the enset farmers as its bleeding (red liquid) when parts are cut. The origin of certainly cultivated enset is evident from the name. One such example in this study is *Kembat* which may be originated from Kembata; however, its name in Kembata-Tembaro and Hadiya is called *Disho* (Table 6).

**Table 6:** Different local (vernacular) names for the same enset plants within or among zones

No.	**Hadiya**	**Kembata-T**	**Silte**	**Gurage**
1	*Shate/Shatedegn*	*Shate*	*Shireteye*	*Shireteye*
2	*Disho*	*Disho*	*Kembat*	*Kembat/Hambediya*
3	*Xiggo*	*Qeqile-nech*	*Dem-worad*	*Dem-yetert nech*
4	*Meqelwesa*	*Qeqile- qey*	*Bushawese*	*Dem-yetert qey*
5	*Bequcho*	*–*	*–*	*Sisasir*
6	*Shereqa*	*–*	*Megrib*	*Yeqisew/Qesew*
7	*Soqido/Soqe*	*–*	*Kemele*	*Kemele*
8	*Qombotira*	*–*	*Ashaqit/Kombotir*	*Ashaqit*
9	*Dego*	*Degomerza*	*–*	*–*
10	*Merza*	*Abatemerza*	*Merza*	*Merza*
11	*Boshosha/Qebere*	*Xebere*	*Tem-wese*	*–*

In addition, according to farmers, some landraces were named based on the color of pseudostem and leaf (*Bushawese* in Silte meaning red enset), but this landrace in Hadiya is given the name *Meqelwesa*, meaning placental enset, which is related to use characters. Similarly, landraces *Soqido* is salt (taste of boiled corm or *amicho*) in Hadiya while in Silte and Gurage it is *Kemele* meaning Ape (maybe the color of the pseudostem or petiole) (Table 6). In general, this is observed due to the use of various local names in the different communities of the study area, having their specific characters and method of perceiving by the local farmers. Based on key informants' responses and focus group discussion, some cultivated enset landraces were named with minor or slight dialect differences forms in the local names among study zones. Those include landraces: *Gimbo*/*Gimbuwa*, *Hiniba*/*Heniwa*/*Enba*, *Jegirada*/*Z’girad*, *Hyro*/*Ahiro*, *Qibnare*/*Qinare*/*Qiniwara,* and *Guary*/*Gariya*. This reveals that sometimes the same landraces are often known by different names in different or the same regions. The method of the naming of landraces as indicated by farmers in our study is also similar to what has been reported in other enset-growing zones. For instance, [12, 15], and [22] reported that the naming criteria of some enset landraces in the Wolaita, Sidama, and Ari respectively, are mostly based on morphological and agronomic traits, place of origin, various uses, and culinary attributes. In the study areas, farmers use their local language in everyday speech and communication in each zone. There are numerous enset landrace names and synonyms in these different languages and dialects were recorded throughout the study zones (Table 5). For instance, in this study 15 identically named enset landraces were identified from all four studied zones. In the same manner, three zones (Hadiya, Silte, and Gurage) commonly share 33 of the same named enset landraces in the present study. A similar observation was notified by [12, 13], and [15], they also described the existence of identically named enset landraces in more than one ethnolinguistic community. This may occur due to getting the enset planting materials and a long-lasting practice of farmers in sharing with their respective landrace names from adjacent administrative zones. Similarly, [12] stated the presence of 'borrowed' landrace names between ethnolinguistic groups. Similar trends were also observed in different traditional crops such as sorghum [11], banana [26], sweet potato [27], cassava [41], and common bean [42, 43]. Our study has also shown that enset growers sometimes delivered various names for the same landrace within the zones. For instance, the landrace named *Ayase* is known as *Hella* in Hadiya Duna *woreda*, *Qombotira* is called *Asheqit* in Silte, and also *Gegered* is known as *Heniwa* in Endegegn *woreda* of Gurage zone. Bareke et al. [42] and Abera et al. [43] also reported similar results from Ethiopia for common beans. Likewise, the different names for the same enset landrace also exist among zones (e.g., *Disho* in Hadiya and Kembata-Tembaro is known as *Kembat* in Silte and Gurage, *Tem*-*wese* in Silte is also called *Xebere* in Kembata-Tembaro or *Qebere* in Hadiya) (Table 6). Moreover, the names of some enset landraces have the same meaning but it was locally known with different folk names throughout study zones. For instance, *Xiggo* in the Hadiya, *Dem*-*worad* in the Silte and *Demyetertqey* in the Gurage refer to bleeding because of exuding red fluid when any part of the enset is cut. This is similar to the findings of [43] who found that common bean producers provided different names in terms of seed color in two areas but the names have the same meaning.

### Pattern of use and management practices undertaken by farmers

Traditionally, farmers in the study area were familiar with the utilization and management of enset from earlier generations to meet their food, drug, and other requirements. In the study area, all enset landraces were primarily cultivated for food and feed use, except landrace *Meqelwesa* or *Qeqile*-*qey* which was rarely used as food. This landrace is one of the most traditionally preferred medicinal enset landraces recommended for human and cattle ailments (Table 7). Based on the information we acquired during the individual interview and focus group discussion, enset farmers preferred landraces with early maturity and vigorous growth, easily harvestable, early fermenting, high *qocho* and *bulla* yielding, and good cooking qualities. In addition, in all four zones, generally, multi-use enset landraces were highly chosen and more cultivated than specific-use landraces. However, in some situations, there was regional or ethnic preference across the study zones.

According to a result of the key informants ranking from the five commonly shared and the other two, *Gimbo* became the first, *Separa* the second, and *Agade* the third most preferred enset landraces for their *qocho* and *bulla* quality; *Astara* and *Agade* scored the highest points for both their *amicho* (cooked corm) tasty and medicinal value, and *Sisqella*, *Bededede,* and *Gimbo* stood first to third, respectively for their fiber quality (Table 8). For instance, extracting *bulla* from other harvested masses of enset (Fig. 2e) in Gumer *woreda* of Gurage zone by women is not common practice, unlike other *woredas* and zones. But they purchase it from other adjacent *woreda* markets for different purposes. In the same pattern, the use and production of fiber, which is another enset product obtained from the decorticating of petiole and pseudostem are decreasing in most of the studied zones. Because it employs a traditional production method that requires more time and labor. In addition, nowadays most of the traditional fiber-made products are replaced by other plastic materials. However, some enset farmers in Hadiya and Kembata-Tembaro preferred more droughts tolerant and high fiber quantity and quality (Table 9) in addition to *qocho* and *bulla* yield, while those in Gurage and Silte favored easy harvesting and processing, early fermenting, and less fibrous landraces (Table 10). The present study also indicated that there were slight differences in terms of perceiving enset end-users across the study zones.

**Table 7:** Enset landraces selected for medicinal purposes

Admin. Zone	Landraces	*N* = 60	Product uses to treat ailment
Hadiya	*Agade*	38	*Amicho* with yoghurt to cure bone fracture
	*Astara*	48	*Amicho* with milk to cure bone and muscle problems in human
	*Bedededa*	35	*Amicho* to initiate milk production in cattle
	*Gishira*	60	*Amicho* and roasted *bulla* with milk to treat bone fracture, in humans and corm to cure broken bone in cattle
	*Hayiwona*	45	*Amicho* with yoghurt to remove spines and swells with pus from the human body, and to initiate milk production in human and cattle
	*Meqelwesa*	60	*Amicho* for human, leaf, and pseudostem for cattle to discharge delayed placenta after birth
	*Qiniwara*	50	*Amicho* with dairy products to cure bone problems in human
	*Qombotira*	32	*Amicho* with yoghurt to treat muscular cramps and waist problem in human
	*Xessa*	42	*Amicho* with milk is eaten to relief broken bone in human
	*Xiggo*	48	*Amicho* to cure kidney problems and hepatitis
K T^*^	*Astara*	38	*Amicho* to treat bone problems in human
	*Cherquwa*	56	*Amicho* with dairy products to remove spines and swells from human body
	*Gishira*	58	*Amicho* and roasted *bulla* with dairy products to treat bone problem in human and raw corm to heal broken bone in cattle
	*Qeqile-nech*	46	*Amicho* for aborification purposes and to treat kidney problem
	*Qeqile-qey*	60	*Amicho* to remove delayed placenta after birth in human, and pseudostem and leaf for the same purpose in cattle
	*Wolagella*	36	Water squeezed from pseudostem to treat skin problem in human
	*Xessa*	58	*Amicho* with dairy products to cure broken bone in human
Gurage	*Astare*	60	*Amicho* with milk to treat bone and muscle problems, and for the initiation milk production in human after delivery
	*Dare*	41	*Amicho* to cure damaged parts of the human body
	*Demyetert*	45	*Amicho* with milk to remove delayed placenta in human
	*Guary*	56	*Amicho* with milk to heal bone fracture in human
	*Oret*	39	*Amicho* with dairy products to expel swells from human body
	*Qibnare*	60	*Amicho* with cheese or yoghurt to treat broken bone and lung diseases in human
Silte	*Agade*	47	*Amicho* with milk to cure bone problems of human and cattle
	*Ashaqite*	38	*Amicho* with yoghurt to treat waist problem in human
	*Astare*	60	*Amicho* with dairy products to repair broken bone, muscles, and to initiating milk production in human
	*Dem-worad*	55	*Amicho* with milk to remove delayed placenta, to cure kidney and liver problem in human
	*Deriye*	43	*Amicho* to heal damaged parts of the human body
	*Guary*	56	*Amicho* with milk to cure bone fracture
	*Hayiwogna*	48	*Amicho* with yoghurt to expel swells and any spiny materials from human body
	*Qiniware*	60	*Amicho* with dairy products to treat broken bones, muscle and lunge problems in human
	*Sino*	42	*Amicho* with dairy products to expel swells from human body

K-T* =Kembata-Tembaro

**Table 8:** Preference in direct matrix ranking of five commonly shared and other two localized enset landraces cultivated in southern Ethiopia

Use value	Landrace	R1	R2	R3	R4	R5	R6	R7	R8	R9	R10	R11	R12	Total	Rank
*Qocho* & B^*^ quality	*Agade*	7	8	7	6	7	6	8	8	7	9	8	9	90	3
	*Astara*	8	7	7	6	6	7	7	8	7	8	7	7	85	4
	*Bededed*	5	6	7	5	4	6	6	7	8	7	8	7	76	5
	*Gimbo*	10	9	10	9	8	10	9	8	7	8	7	8	103	1
	*Gishira*	4	3	3	4	5	4	0	0	0	0	0	0	23	7
	*Separa*	8	9	8	9	9	7	8	8	9	9	8	8	98	2
	*Sisqella*	6	5	6	7	6	7	6	0	0	5	4	0	52	6
*Amicho* tasty	*Agade*	7	8	8	6	7	7	8	9	8	9	7	9	93	2
	*Astara*	10	10	10	10	10	10	10	10	10	10	10	10	120	1
	*Bededed*	1	1	1	1	1	1	1	1	1	1	1	1	12	5
	*Gimbo*	6	7	6	7	5	6	6	5	6	7	5	6	72	4
	*Gishira*	1	1	1	1	1	1	0	0	0	0	0	0	6	7
	*Separa*	7	6	7	7	7	6	7	7	6	7	7	7	81	3
	*Sisqella*	1	1	1	1	1	1	1	0	0	1	1	0	9	6
Fiber quality	*Agade*	5	5	4	3	6	4	6	5	7	5	5	6	61	5
	*Astara*	4	5	4	3	4	3	5	6	3	6	4	3	50	6
	*Bededed*	7	6	7	6	6	7	7	7	6	8	6	7	80	2
	*Gimbo*	5	7	7	4	7	6	7	7	6	7	7	6	76	3
	*Gishira*	8	9	8	8	7	9	0	0	0	0	0	0	49	7
	*Separa*	5	6	6	7	5	4	7	4	5	6	6	4	65	4
	*Sisqella*	10	10	10	10	10	10	10	0	0	10	10	0	90	1
Medicinal use	*Agade*	7	6	8	4	5	3	8	7	9	6	7	7	77	2
	*Astara*	10	10	8	7	8	9	10	10	10	10	10	10	112	1
	*Bededed*	8	8	6	6	7	6	6	7	6	6	4	6	76	3
	*Gimbo*	6	3	2	4	3	1	3	1	3	1	2	3	32	6
	*Gishira*	10	10	10	10	10	10	0	0	0	0	0	0	60	4
	*Separa*	5	4	4	6	5	4	3	2	1	4	2	1	41	5
	*Sisqella*	3	2	1	2	1	1	1	0	0	1	0	0	12	7

R for respondents, 10 for most valuable, 1 for least valuable, and 0 for the uses not known R1-R3 from Hadiya, R4-R6 from Kembata-Tembaro, R7-R9 from Gurage and R10-R12 from Silte zones; B* for *bulla*

**Table 9:** Enset landraces selected for strong and long fiber

Hadiya zone		Kembata-T zone		Silte zone		Gurage zone	
Landraces	*N* = 60	Landraces	*N* = 60	Landraces	*N* = 60	Landraces	*N* = 60
*Sisqella*	60	*Sisqella*	60	*Kembat*	52	*Kembat*	53
*Disho*	56	*Gishira*	57	*Bededet*	50	*Yeshirenqinke*	49
*Unjame*	54	*Unjame*	55	*Gimbo*	41	*Bededet*	48
*Gishira*	55	*Disho*	48	*Separa*	40	*Gimbuwa*	40
*Dirbo*	42	*Dirbo*	41	*Agade*	35	*Sebara*	38
*Dego*	40	*Shelleqe*	39				
*Bequcho*	39	*Hella*	38				
*Bedededa*	36	*Degomerza*	37

**Table 10:** Enset landraces selected by farmers for *amicho*

No.	In Hadiya zone		In Kembata-T		In Silte zone		In Gurage zone	
	Landrace	*N* = 60	Landrace	*N* = 60	Landrace	*N* = 60	Landrace	*N* = 60
1	*Soqido*	52	*Leqeqe*	58	*Qinare*	60	*Qinare*	60
2	*Qiniwara*	51	*Xebere*	51	*Astare*	60	*Astare*	60
3	*Astara*	51	*Quena*	50	*Gariye*	57	*Guarye*	58
4	*Gariya*	47	*Xorore*	50	*Ashaqit*	50	*Kemele*	43
5	*Leqeqe*	39	*Astara*	46	*Agade*	48	*Ginad*	35
6	*Xorore*	38	*Sebara*	36	*Oret*	36	*Oret*	36
7	*Quena*	37	*Etene*	35	*Torore*	35	*Torore*	37
8	*Qombotira*	37					*Qesew*	39
9	*Qebere*	36					*Ashaqit*	37
10	*Orada*	35					*Bezeria*	36

Moreover, interviewed farmers in Kembata-Tembaro grouped enset landraces into two major sex categories: female enset and male enset. The division of male and female is not linked to biological reproduction but it is based on perceived features of the landraces. The female groups are known for ease of decorticating, early fermentation, corm palatability, more susceptibility to different diseases, and low strength of fiber whereas the male groups contrast to these characteristics. In contrast, farmers in Hadiya, Gurage, and Silte did not tend to classify enset plants into sex designation. Tsegaye [17] also reported the relationship to the difference in food culture, sociocultural preferences for different enset products, and farming systems of the regions. Similarly, [44] described the influence of cultural background on plant species diversity and the uses of plant species for different purposes. Enset landrace diversity within the same and different cultural groups nicely demonstrates that cultural needs and requirements are key factors in the diversification of crop varieties. In particular, the unique landraces recorded in the different ethnic communities indicate the origin and maintenance of those landraces by specific ethnic groups because they need them for their food, medicine, and other uses.

According to the farmers' report, we identified a total of 32 landraces which were applied in different proportions by each ethnic group: 10 in Hadiya, 9 in Silte, 7 in Kembata-Tembaro, and 6 enset landraces in Gurage as traditionally medicinal use to treat various health problems in human and cattle (Table 7). Out of the total listed, 12 medicinally used enset landraces shared the identical name in at least two zones, so the total number decreased to 20. Landrace like *Astara* mentioned by the farmers is an example of enset that has multiple uses of traditional medicinal purposes in the all study area. Furthermore, landraces such as *Qinare*/*Qiniwara*, *Gishira*, *Guary*, *Xessa*, *Hayiwona,* and *Agade* were also the most frequently used medicinal enset present in homegardens of two or more ethnic communities (Table 7). On the other hand, some medicinal landraces (*Cherquwa* and *Wolegella*) were identified as having narrow distribution in the study zones. However, in some cases the same kinds of enset are known with alternative local names used as medicines for different problems among the study communities (Table 7).

For instance, landrace *Xiggo* in Hadiya is mainly traditionally used to treat kidney and liver problems, whereas the same variety with different names (*Qeqile*-*nech* in Kembata and *Dem*-*worad* in Silte) quoted by many farmers to remove the delayed placenta and for aborification (used to cause/facilitate abortion) purposes (Table 7).

This may be due to each ethnic community having its ways, practice, and beliefs to utilize enset plants. All of the traditionally medicinal enset landraces were also selected for sweet *amicho* (cooked corm) production except landraces *Gishira*, *Dare, and Bedededa.* In the same manner, the most chosen part of enset for medicinal use was corm but the landrace *Meqelwesa* (in Hadiya) or *Qeqile*-*qey* (in Kembata) were all part used as traditional medicine. In some cases, farmers also used cooked *qocho* or porridge prepared from *bulla* to treat different health problems in the study zones (Fig. 2f and g). In terms of connection to the ailments shared by the farmers and the medicinal enset landraces used in their treatment, we observed that bone fracture, swelling of the pus and to expel the delayed placenta from humans and cattle were the most shared health problems in the study area and among the communities. We observed that some ethnic groups (e.g., Silte and Gurage, Hadiya and Kembata-Tembaro) share more medicinal enset landraces and show greater similarity in patterns of using enset crop (Table 7). In the same manner, [44] stated that intercultural sharing may be explained by the pharmacological effectiveness of shared medicinal plants among ethnic groups.

Most enset-growing farmers in the study area are familiar with maintaining and use of their different preferred landraces to stabilize many situations over a long period without external support and inputs of planting materials. Farmers in the study zones frequently produce their planting materials or suckers from homegardens but few farmers obtain them freely from neighbors, family, and friends as a gift or by purchasing from other farmers. This was in line with the reports of [7, 13].

During our discussions with farmers and field observation, we observed that in two local markets: Alicho in Silte and Gumer in Gurage zones, enset suckers were purchased from January to April. These two sites are situated at a higher altitude than other studied *woredas* (Table 1). Moreover, some elder farmers mentioned that enset cultivation practice and its distribution into their *woreda* and villages relatively late than others. They said that “We haven’t been familiar with enset production and managing before 65 years ago.” To some extent, this verifies that enset farming systems in the studied area are not equally and uniformly experienced within and among communities. Enset cultivation and use culture has been gradually and slowly moving to the peripheral areas from region to region, from zone to zone, and from district to district due to farmer “experimentation” and horizontal transfer of indigenous knowledge. In a similar vein, [2, 5], and [18] indicated that the distribution of cultivated enset in Ethiopia appears to be expanding, especially after periods of devastating famines of the 1980s, when people in other regions learn about the benefits of this crop and attempt to incorporate it into their farming system. They have also shown that enset moves some inches into the Oromia region and this is observed in southwest Shewa and southeast Arsi and may be in the western part of Bale. Negash [16] also noted that during the drought period, many farmers migrated from their villages to as far away in search of food, and there they learned about enset production. When they came back to their homesteads, they introduced enset. Furthermore, [45] and [46] show that smallholder farmers expand the production area of the perennial crop enset as a climate coping strategy in a drought-prone indigenous agrisystem.

Farmers in the studied area maintain great enset landraces diversity within traditional cultivation and production systems insight toward meeting domestic subsistence requirements. Yemataw et al. [13] also described that farmers observe and select the landraces based on their planting intentions for the coming year than the proportion to the quantity they have. The on-farm maintenance of biodiversity requires understanding by the farmer of how specific varieties should be grown, stored, and maintained to maximally realize the characteristics these farmers value [47].

## Conclusion

This study provides information on enset landraces existing in four major enset-producing administrative zones of Ethiopia based on local farmers-named landraces in each of the zones. The results obtained from this study indicate that farmers have developed diverse practices and experiences over time to cultivate, utilize, and conserve a great extent of enset landraces in each zone. In addition, they understand the need to grow a mixture of enset landraces as this can have roles in the socioeconomic and cultural life of communities. Our results have revealed that out of the cultivated enset landraces, a small proportion of landraces were widely distributed and abundant throughout the study zones. However, a larger number of landraces were highly localized in one or two studied zones and less distributed in other zones. Our study also confirms that farmers can differentiate their enset landraces by using their different local names. In this context, some enset landraces were commonly known and referred to by the same local names in all studied zones by different farmers. In contrast, enset of the same landraces were named differently by different farmers within and among studied zones. Moreover, results from this study also show that enset farmers have developed their way of selecting and characterization of landraces with some slight differences among them in terms of use patterns based upon their traditions and cultures in the study areas. Based upon the results of this study, the on-farm diversity existing in these landraces needs to be studied in detail (e.g., molecular characterization) for duplicates identification and clarification of synonymies, and to facilitate their on-farm conservation as well as sustainable utilization of enset farming communities and also in its improvement programs. A new study shows that frequent severe drought events led to an increase in enset production areas in Ethiopia. Indigenous staples are “saviors” during difficult times. This is why national investment in their conservation, improvement, and value addition is necessary for a changing climate.

## Data Availability

All data generated or analyzed during this study are included in this manuscript.
